# Drug review: mTOR-inhibitor therapy in fetal cardiac rhabdomyoma—a tightrope walk

**DOI:** 10.3389/fped.2025.1649341

**Published:** 2025-08-26

**Authors:** Nadine Muschel, Michaela Höck, Elke Griesmaier, Samira Abdel Azim, Elisabeth Ralser, Christina Schreiner, Elisabeth Schermer, Ursula Kiechl-Kohlendorfer, Irene Mutz-Dehbalaie, Miriam Michel

**Affiliations:** ^1^ Department of Obstetrics and Gynaecology, Medical University of Innsbruck, Innsbruck, Austria; ^2^ Department of Paediatrics II (Neonatology), Medical University of Innsbruck, Innsbruck, Austria; ^3^ Department of Paediatrics III (Cardiology, Pulmonology, Allergology and Cystic Fibrosis), Medical University of Innsbruck, Innsbruck, Austria

**Keywords:** adverse effects, fetal cardiac tumor, rhabdomyoma, sirolimus, re-entry tachycardia

## Abstract

**Objective:**

Mechanistic/mammalian target of rapamycin (mTOR) inhibitors have been used successfully to reduce the size of cardiac rhabdomyomas. However, the number of published cases is small and thus there is no consensus about therapeutic approaches, especially regarding dosing regimens and safety profiles of mTOR inhibitors. Based on a systematic literature review and one new case report, we discuss in detail the indication and adverse effects of fetal and neonatal mTOR-inhibitor therapy.

**Methods:**

A comprehensive search was conducted on PubMed/MEDLINE and Web of Science for studies using combinations of the relevant medical subject heading (MeSH) terms and keyword (rhabdomyoma AND fetal OR fetus OR prenatal AND cardiac AND sirolimus) from the first report in 2018 until July 2025. Studies were included if they reported on pregnancies with fetal cardiac tumor and rhabdomyoma entity suspicion treated with mTOR inhibitors.

**Results of literature review and new case description:**

In total, 67 results were found. After excluding non-eligible publications, a total of 20 documented cases were identified from 15 reports, all presenting lifesaving effects of mTOR inhibitors in fetuses and neonates with cardiac rhabdomyomas. We report on a patient with a prenatally suspected cardiac rhabdomyoma, which, due to imminent bilateral outflow tract obstruction, was prenatally treated with sirolimus. Tumor regression could be achieved. For maternal medical reasons, prenatal sirolimus had to be stopped after 5 weeks. Postnatal incessant atrioventricular re-entrant tachycardia occurred, which was unresponsive to electric or medical cardioversion (amiodarone) and unresponsive to everolimus. The patient developed massive capillary leak syndrome within hours. In combination with restrictive ventricular filling properties, the tachycardia resulted in death on the seventh day of life.

**Conclusion:**

Cardiac rhabdomyomas have the potential to become a life-threatening condition, not only by impairing myocardial function and cardiac outflow, but also by causing arrhythmia due to tumor muscle bundles as substrate for a pre-excitation syndrome resulting in intrauterine or postnatal atrioventricular re-entrant tachycardia, as observed in our patient. The pharmacological therapeutic approach is fetal and neonatal treatment with mTOR inhibitors. All previous reported cases present lifesaving effects of mTOR inhibitors in fetuses and neonates with cardiac rhabdomyomas; however, adverse effects cannot be disregarded.

## Introduction

Cardiac rhabdomyoma, though generally rare, represents the most prevalent primary cardiac tumor in the fetal population, constituting 60%–86% of all primary fetal cardiac tumors (1). It has a strong genetic association with tuberous sclerosis complex (TSC), occurring in 80%–90% of cases and rising to as high as 95% when lesions are multiple or there is a positive family history (2). It has a rather benign course, is generally noted in the second trimester, and often grows until 30–32 week of gestation, with spontaneous intrauterine or postnatal regression (3). In rare instances, it is associated with (bilateral) ventricular outflow tract obstruction, impaired myocardial function, and/or arrhythmia, with low cardiac output and congestion, with incipient hydrops and fetal demise (4).

Therapeutic approaches with mechanistic/mammalian target of rapamycin (mTOR) inhibitors (such as everolimus and sirolimus) have been used successfully postnatally to reduce the size of cardiac rhabdomyoma (5, 6). The first reports of intrauterine treatment via transplacental mTOR inhibitor administration emerged in 2018 (7). However, the number of published cases is small and the dosing regimen and safety profile of sirolimus and everolimus remain undefined (7–22). All previously reported cases highlight the lifesaving effects of mTOR inhibitors in fetuses and neonates with cardiac rhabdomyomas (23). The aim of this paper is to synthesize current evidence on transplacental mTOR-inhibitor use, focusing on dosing strategies, maternal–fetal safety, and clinical decision frameworks, and to detail a novel case of fetal sirolimus therapy complicated by prenatal maternal side effects and early therapy discontinuation.

## Methods

**Figure 1:**
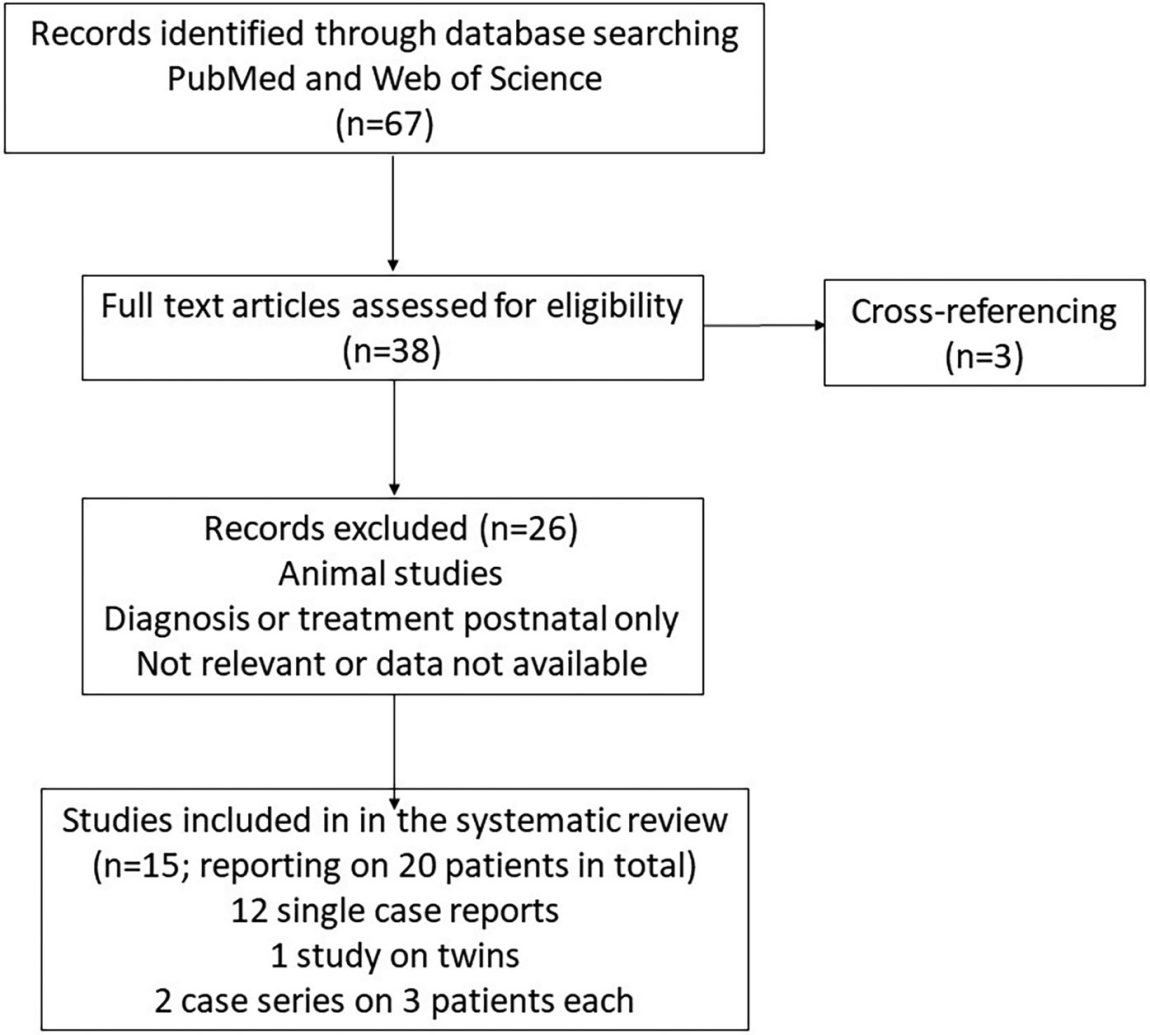
PRISMA flowchart.

Our work is a hybrid: (1) synthesizing current evidence on transplacental mTOR-inhibitor use, focusing on dosing strategies, maternal–fetal safety, and clinical decision frameworks [systematic review performed according to the Preferred Reporting Items for Systematic Reviews (PRISMA) guidelines]; and (2) presenting a new case on transplacental mTOR-inhibitor therapy entailing maternal complications with the need for early therapy discontinuation. We searched PubMed (https://pubmed.ncbi.nlm.nih.gov) and Web of Science, from the first report in 2018 until July 2025. The literature search was conducted using combinations of the relevant medical subject heading (MeSH) terms and keyword (rhabdomyoma AND fetal OR fetus OR prenatal AND cardiac AND sirolimus). The inclusion criteria were full-text articles reporting on pregnancies with suspected fetal cardiac tumors of the rhabdomyoma type that were treated with mTOR inhibitors. A total of 67 results were found. In total, 20 documented cases were identified from 15 reports [nine single patient reports (7, 9, 11–13, 15–17, 21), one report of twins, thereof only one with relevant tumor size (22), two case series with three patients each (10, 24); three additional single case reports were identified by cross-referencing (14, 18, 20)]. The exclusion criteria were studies in which cardiac rhabdomyoma was diagnosed or treated only postnatally, and those that did not report individual background data for the included cases. The PRISMA flowchart is shown in Figure 1. Clinical findings, outcomes, and dosing regimens from previously reported cases involving prenatal mTOR-inhibitor therapy for cardiac rhabdomyoma were reviewed, tabulated, and discussed.

## Novel case—maternal side effects

A 38-year-old gravida 2 woman with genetically confirmed TSC (subunit 2: c.1946 + 1G > A), epilepsy, and mild intellectual impairment—whose first child is also affected—was referred to the Fetal Medicine Unit at the Medical University of Innsbruck, Tyrol, at 22 weeks of gestation due to the detection of a large fetal cardiac tumor on routine prenatal ultrasound.

**Figure 2:**
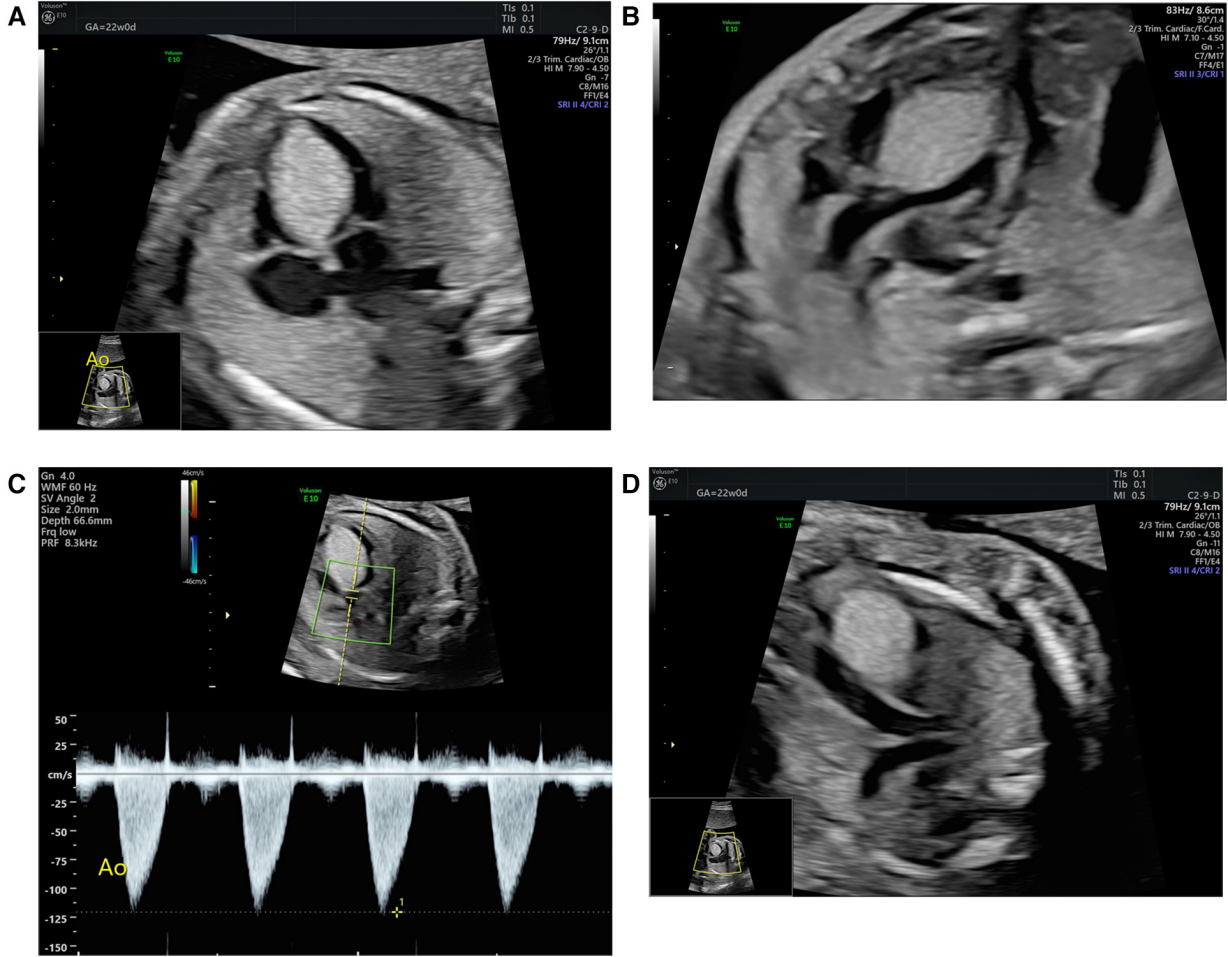
Fetal echocardiography before transplacental sirolimus therapy initiation: apical four-chamber view displaying the large hyperechogenic tumor mass (yellow markers) located in the interventricular septum, extending from the atrioventricular region to the apex and reaching into both chamber cavities (**A**). The modified five-chamber view (**B**) displays the imminent obstruction of the left ventricular outflow tract by the tumor mass, with a respectively altered Doppler flow pattern across the left ventricular outflow tract showing moderately increased flow velocity (**C**). The modified vessel plane (**D**) shows the imminent obstruction of also the right ventricular outflow tract by the tumor mass.

At initial evaluation, a 12×15 mm echogenic mass was identified arising from the interventricular septum and projecting into the left ventricle, without outflow obstruction. One week later (at 23 weeks of gestation), the mass had grown to 16×16 mm, extending into the right ventricle and causing imminent bilateral outflow tract obstruction (Figure 2A–D). No additional lesions, malformations, or functional abnormalities, such as arrhythmia or hydrops, were suspected.

The case was reviewed by our multidisciplinary perinatal board. Given the progression of the cardiac mass and emerging subobstruction of the aortic outflow (V_max_ 110 cm/s, normal ≤ 100 cm/s; pulmonary outflow at that time unobstructed with V_max_ 90 cm/s), along with the early gestational age, anticipated further tumor growth, and septal location with potential bilateral obstruction—limiting postnatal intervention via an open duct (25)—we decided to initiate transplacental treatment with sirolimus, an mTOR inhibitor.

At 23 + 2 weeks of gestation, maternal oral sirolimus was initiated with a loading dose of 6 mg, followed by 2 mg once daily. Maternal whole blood sirolimus levels were monitored weekly. Initial blood sirolimus levels were low; therefore, the dose was increased stepwise up to 16 mg once daily, aiming at a whole blood sirolimus level of 10 ng/ml (7–10). Daily oral administration of 16 mg sirolimus resulted in a maternal trough level of 8.1 ng/ml. After 3 weeks of treatment, the tumor showed no further increase in size.

**Figure 3:**
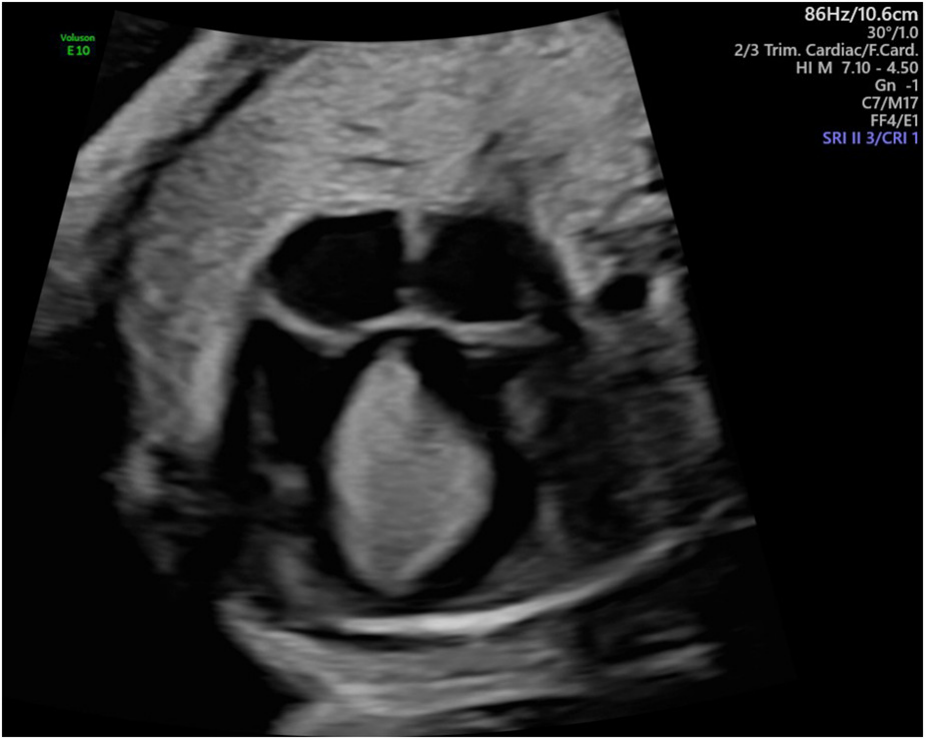
Fetal echocardiography at the time of cessation of transplacental sirolimus therapy: four-chamber view showing the septal tumor mass, now slightly reduced in size.

After 5 weeks of sirolimus treatment, a decrease in absolute tumor size was observed (Figure 3), and V_max_ had normalized to 95 cm/s for the aorta and remained at 90 cm/s for the pulmonary artery.

**Figure 4:**
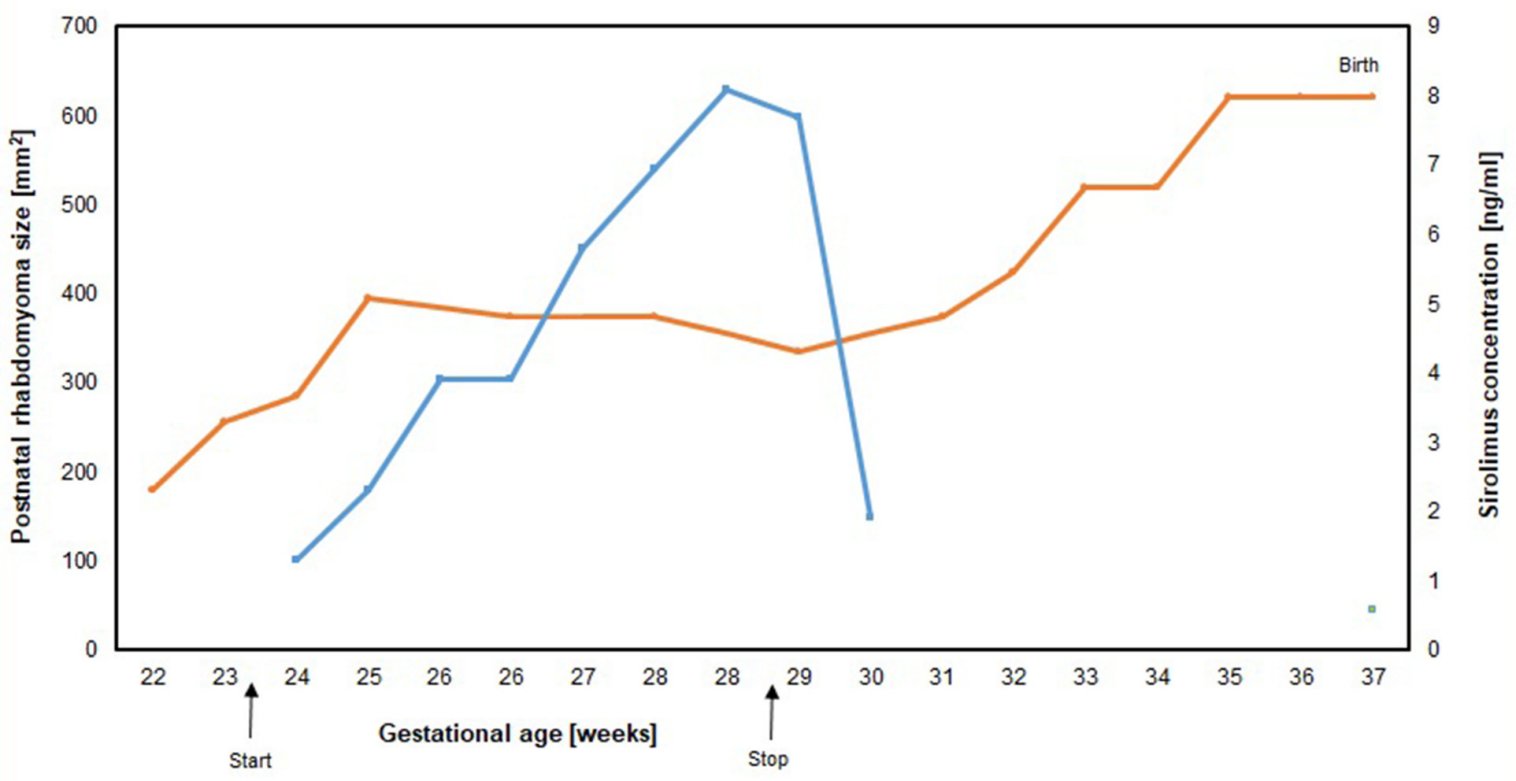
Rhabdomyoma size (orange line) measured by fetal echocardiography in the apical four-chamber view and maternal sirolimus blood level (blue line) and sirolimus cord blood level (green square) by 37 weeks of gestation). The points on each line denote individual echocardiographic measurements of the area of the tumor mass, arrows denote the initiation and the discontinuation of transplacental sirolimus treatment.

Weekly checks of maternal blood chemistry, including blood count, lipid status, and infection parameters, remained normal. After 2.5 weeks of sirolimus treatment, the mother reported a productive cough without any clinical or laboratory findings suggestive for infection. The productive cough intensified with increasing doses of oral sirolimus. We were concerned about the mother's risk of developing serious interstitial lung disease (pneumonitis) as she lived and worked on a farm. To further investigate this possible side effect, a chest radiograph/computed tomography (CT) scan was recommended but was declined by the patient, and sirolimus treatment was terminated at 28 + 4 weeks of gestation, i.e., 5 weeks after initiation. One week after sirolimus termination, the maternal cough resolved. The fetal cardiac mass remained stable in size until term, when a planned cesarean delivery for maternal indication at 37 + 3 weeks of gestation was performed (Figure 4). One week before delivery, computerized cardiotocography (CTG) documented a self-limited fetal tachycardia with 190–200 bpm over a period of 5 min. On ultrasound, there were no signs of hemodynamic compromise or fetal hydrops with normal extracardiac fetal Dopplers. The male neonate presented with an APGAR score of 6/8/9, an umbilical artery pH of 7.27, a base excess of −0.6 mmol/L, and a birthweight of 3,600 g (82nd percentile). His cord whole blood level of sirolimus was <0.6 ng/ml (9 weeks after cessation of sirolimus, level compatible with the terminal half-life of sirolimus in adults being 62 ± 12 h) (26).

**Figure 5:**
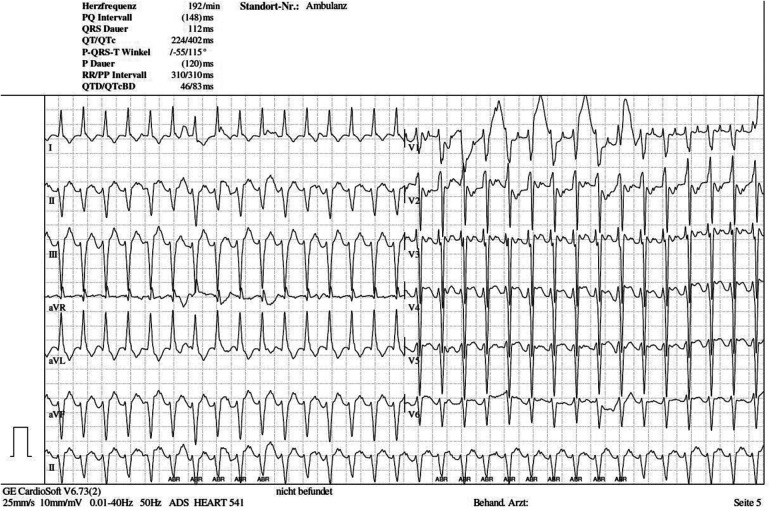
Electrocardiogram 1 h postnatally: broad complex re-entrant tachycardia with retrograde p wave.

From the time of birth, the patient was in severe respiratory distress and underwent resuscitation with positive pressure ventilation up to pressures of 30/5 cm H_2_O (PIP/PEEP) and a FiO_2_ of 40%. On his electrocardiogram (ECG), the neonate showed a regular broad complex re-entrant tachycardia, with a maximum rate of 220 bpm (Figure 5). There was a retrograde p-wave present. Intravenous adenosine (3 × 0.7 mg) and electric cardioversion (1 J/kg bodyweight) resulted in only transient termination of the re-entrant tachycardia, rendering the tachycardia incessant by definition. Over the first 5 h, the infant’s respiration deteriorated, with increasing oxygen demand (FiO_2_ of 100%). The infant was then intubated, and a total of 720 mg (200 mg/kg) of surfactant (Curosurf®) was administered. However, his respiration stabilized only after switching to High Frequency Oscillation ventilation.

**Figure 6:**
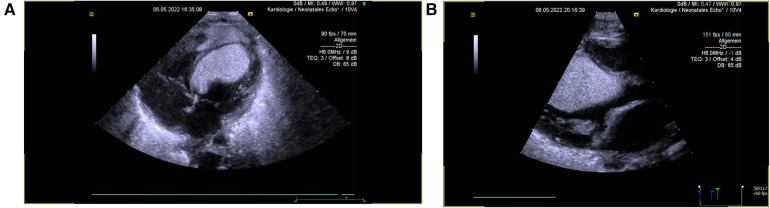
Echocardiography 2 h after birth: apical four-chamber view (**A**) and parasternal long axis (**B**) showing one very large tumor in the LV cavity, originating from the septum (30.5 × 18 mm^2^).

Regarding the cardiac mass located in the interventricular septum affecting the atrioventricular junction (Figure 6A,B), the tachycardia was regarded as likely reflecting a re-entrant mechanism due to tumor mass muscle bundles (27, 28). Ventricular tachycardia was considered a critical, albeit unlikely, differential diagnosis, potentially attributable to mTOR-inhibitor toxicity (considered unlikely given a low cord blood sirolimus level of <0.6 ng/ml), infection (ruled out by negative laboratory findings: CRP < 0.06 mg/dl and IL-6 20.1 ng/L, though antibiotic therapy was started), or an idiopathic cause. A wide QRS reciprocating atrioventricular tachycardia could not be excluded. After continuous intravenous application of amiodarone (total cumulative dose: 303 mg, 84 mg/kg) and esmolol (total cumulative dose: 182 mg, 51 mg/kg), heart rate control was successfully achieved the same day, at an acceptable range of 160–180 bpm. On the second day of life, mTOR-inhibitor therapy was reintroduced. According to the current literature, oral everolimus was administered (29–32). The starting dose was 1 mg, followed by a maintenance dose of 0.5 mg given every 12 h (4.5 mg/m^2^/day), with a target trough level of 5–15 ng/ml (29). On the third day of life, short periods of rhythm control were achieved; however, the neonate soon developed massive capillary leak syndrome, leading to refractory arterial hypotension that needed inotropic support. Although peripheral and generalized edema as well as hypoalbuminemia have been reported with everolimus, neither tachyarrhythmia nor arterial hypotension have been previously described for either everolimus or sirolimus (33–37). To minimize the risk of proarrhythmogenic or tachygenic medication, adrenaline was initially avoided, and norepinephrine was used instead. However, progressive ventricular function and persistent hypotension required the addition of hydrocortisone, as well as adrenalin and milrinone combined with norepinephrine. Unfortunately, the massive capillary leak did not resolve in time, and the neonate’s hemodynamic status remained severely compromised. Adequate oxygenation, CO_2_ elimination, and blood pressure stabilization could not be achieved despite the escalated medication and intensive care interventions.

On his seventh day of life, the boy died in his parents' arms as a result of severe multiorgan failure. Tissue samples (oral mucosa DNA) were collected for genetic testing but have not yet been analyzed. Although no cerebral lesions were detected, the presence of both maternal and first child diagnoses of TSC, along with the cardiac lesions observed in the infant, strongly suggests a diagnosis of fetal/neonatal TSC.

## Results and discussion of the review of the literature

Fetal cardiac rhabdomyoma is the most common primary fetal tumor of the heart, accounting for 60%–70% of all heart tumors and is closely associated with TSC (3). The tumor is typically benign and involves either the left or right ventricle and/or the ventricular septum; in 90% of cases, the tumor is multilocal. Cardiac rhabdomyoma can cause outflow tract obstruction, arrhythmias, low cardiac output, hydrops, and, if progressive, heart failure and fetal demise (4).

In our case, the family history, genetic background, tumor location, and clinical presentation strongly suggested rhabdomyoma, although postmortem or genetic confirmation was not available.

The natural course of cardiac rhabdomyomas is regression. However, significant hemodynamic compromise is possible, and therapy with mTOR inhibitors such as sirolimus can be considered. Data on maternal sirolimus in pregnancy or postnatal treatment are sparse. Our literature search identified 67 results. After excluding non-eligible publications, a total of 20 documented cases were identified from 15 publications, all presenting the lifesaving effects of mTOR inhibitors in fetuses and neonates with cardiac rhabdomyomas and suspicion of rhabdomyoma (Table 1).

**Table 1:** Sirolimus treatment for cardiac rhabdomyomas.

Study		GA [w + d]			Sirolimus		
	Indication for treatment	Treatment initiation	Treatment termination	Birth	Dosing (mg/d)	Blood level (ng/ml)	Success of treatment
Barnes et al. (7)	Bilateral outflow tract obstruction SVT Impending fetal hydrops	30	36	36	12 (6.3 mg/m^2^) during first 48 h, additional 22 (11.7 mg/m^2^); then 13 ± 2 (6.8 ± 1.04 mg/m^2^/day)	Maternal target trough level: 10–15 Mother p.p.: 6.9 Cord blood: 11.3	After 3 weeks: tumor regression At 4.5 months: tumor progression after sirolimus discontinuation and regression after restarting At 9 months: continue to receive sirolimus, appropriate somatic growth and development
Park et al. (9)	Pulmonary LAM in mother (therapy already before pregnancy) No fetal indication	23	39	39	4 loading dose 12	Mother p.p.: 25.0 Cord blood: 33.2	After 19 days: number and size of tumors markedly decreased At 29 + 5: no cardiac mass
Vachon-Marceau et al. (11)	Biventricular diastolic and systolic dysfunction Tricuspid regurgitation Pericardial effusion	31 + 4	36	39	15 loading dose 5–8	Maternal target trough level: 10–15	After 4 weeks: mass shrank, ventricular function improved (left ventricular ejection improved from 18 to 33% and right ventricular ejection fraction from 28 to 46%), tricuspid regurgitation resolved
Pluym et al. (12)	LVOTO, Low cardiac output, mitral regurgitation Pericardial effusion Impending fetal hydrops	28	36	36	10 loading dose 6–10	Maternal trough level: 11.6–18.6 Mother p.p.: 3.4 Cord blood: 3.3	After 2 weeks: decrease in tumor size with less obstruction to flow with a decrease in mitral regurgitation After 4 weeks: normalization of cardiac output, ejection fraction improved to 48.5% After 6 months: stable cardiac rhabdomyomas, meeting her developmental milestones
Ebrahimi-Fakhari et al. (10)	Patient 1: LVOTO	35 + 2	39 + 1	39 + 1	1 for two days, then 3	Maternal trough level: 6.1	Gradual reduction in the size of rhabdomyomas and resolution of left LVOTO At 2 years: delayed expressive speech, on target to his receptive speech and fine and gross motor development
	Patient 2: Tumor encapsulating the left ventricle (incipient hemodynamic compromise)	33 + 1	36 + 6	36 + 6	3	Maternal trough level: 3.7	Mass shrank gradually, no obstruction of the LVOT detected At 21 months: delayed speech, fine and gross motor skills were within normal limits
	Patient 3: LVOTO	34	38 + 6	38 + 6	4 + 2 (∼3.3 mg/m^2^/day)	Maternal trough level: 10.9 ± 1.3	After 2 weeks: tumor reduced, LVOTO improved Growth restriction At 16 months: expressive speech delay, growth progress (55th percentile for weight and length)
Dagge et al. (13)	Renal angiomyolipomas in mother (therapy already before pregnancy) Reintroduced for fetal indication: RVOTO Arrhythmia (not classified)	26	39 (therapy with sirolimus was reduced after birth – 4 mg/d)	39	4–10	Maternal trough level: Target 10–15; max. 16.7	After 2 weeks: tumor regression, cardiac rhythm alternated between sinus rhythm and periods of arrhythmia with frequent extrasystoles Postnatal: persistent normofrequent sinus rhythm
Cavalheiro et al. (17)	Ongoing growth of cardiac and brain lesions		39	39	Everolimus, initial dose of 10	Maternal serum level 8.4	Cardiac lesion decreased, intracranial lesions remained stable At birth: small atrial lesion, several intracerebral tubers and subependymal lesions At 36 months: normal neuropsychomotor development
McLoughlin et al. (14)	LVOTO Ventricular dysfunction Hydrops fetalis	30	36	36	2 mg/m^2^/day divided twice daily	Maternal target trough level 10–12	After 2 weeks: decrease in tumor size with subsequent decrease in hydrops After delivery: inadequate cardiac output due to left ventricular systolic dysfunction Epinephrine, milrinone along with oral everolimus was maintained After 3 months: mass measured 0.9 × 1.5 cm with no LVOTO
Will et al. (15)	RV inflow obstruction	27 + 0	38	39 + 1	4	Maternal trough level: 8.4–9.9 Mother p.p.: 1.2 Cord blood: 1.6	After 2 weeks: reduction in rhabdomyoma size and amelioration of ventricular function Postnatal: partial covering of the tricuspid valve without inflow obstruction, further treatment with everolimus After 2 months: further reduction in tumor size After 13 months: 100% reduction in tumor mass, only small residual (3 mm) at lateral base of tricuspid valve Motor and mental development retarded
Maasz et al. (16)	Ongoing growth of cardiac and brain lesions		Duration of 5 weeks	Term	Everolimus, initial dose of 10 and adjusted from to 5 from day 10	Maternal target trough level 5–15	Reduction in tumor size After 4 months: West syndrome (seizures, conculsions, infantile spasms, hypsarrhythmia on EEG) At 1 year: age appropriate values (IQ > 100) of BSID At 36 months: 6–10 months delay in all parameters
Schenk et al. (20)	Progression of intracardiac mass and pericardial effusion	33 + 1	Therapy with everolimus was continued after birth 1 × 0.1 mg (target 5–15)	40 + 4		Maternal target trough level: 10–15 Mother p.p.: 11.6 Cord blood: 5.4	At birth: tumor size decreased Postnatally: Everolimus After 1 year: cardiac and neurological development was unimpaired
Griesman et al. (18)	RVOTO	23	34	37 + 6			After 4 weeks: tumor decreased in size After 2 months: tumor increased in size, recurrence of RVOTO Sirolimus was initiated again and over the next 4 months the tumor nearly disappeared
Bakos et al. (24)	3 patients: Arrhythmias, LVOTO						Obstruction was resolved
Uno et al. (21)	Severe heart failure LVOTO	33 + 3		39 + 2	4	Maternal target trough level: 5–12	After 2 weeks: decrease of tumor size
Ritter et al. (22)	2 patients (twins): Twin B with giant mass with resulting LVOTO and hydrops (twin A with only minimal tumors without indication for treatment)	28 + 3		34 + 2	6		Twin B: After 4 weeks hydrops reversed (Twin A with minor residual tumor masses, no adverse effects through treatment for sibling-only indication)
Muschel et al. (2025) (This article)	Bilateral outflow tract obstruction	23 + 2	28 + 4	37 + 3	Loading dose 6, followed by 2, increased up to 16	Maternal target trough level: 10 Mother p.p: 7.7 Cord blood: < 0.6	After 3 weeks: no further increase in tumor size After 5 weeks: decrease in absolute tumor size, outflow tract obstruction had regressed After 2 days pn: Everolimus After 7 days pn: severe multiorgan failure

The table summarizes the results of the systematic literature review, including reports on 20 patients, plus the new case presented in this publication, bringing the total to 21 cases. Dosing, sirolimus medication applied; level, sirolimus blood trough level, gestational age; SVT, supraventricular tachycardia, LAM, lymphangioleimoymatosis; LVOTO, left ventricular outflow tract obstruction; RV, right ventricle; RVOTO, right ventricular outflow tract obstruction; pn postnatal; pp, postpartal; BSID, Bayley Scales of Infant and Toddler Development; IQ, intelligence quotient.

In 10 reports, the indication for initiating mTOR-inhibitor treatment was progressive rhabdomyoma growth (16–18, 20, 21, 24), mostly with consecutive in- or outflow tract obstruction and imminent low output, congestion, and hydrops fetalis (7, 10, 12–14, 22). Only one case showed bilateral outflow tract obstruction, as seen in our patient (7). Significant prenatal arrhythmias were the indication for treatment initiation in three reports (7, 13, 24); in another two cases, the mother had already been treated with sirolimus before pregnancy because of lymphangioleiomyomatosis (9) (there was no incipient fetal compromise) or for renal angiomyolipomas, where medication had to be restarted because of an increase in tumor mass with mild obstruction of the right ventricular outflow tract and arrhythmia (SVT) with incipient fetal cardiac failure (13). Under sirolimus treatment, there was a significant reduction in tumor size and resolution of the arrhythmia was achieved.

In the published reports, the decision for intrauterine treatment was made at 23–35 weeks of gestation. Sirolimus was applied in all cases except two, where everolimus was introduced (16, 17). The doses applied were in the range of 3–12 mg daily, with varying maternal serum trough levels. Table 1 gives an overview of all available literature on the use of mTOR-inhibitor therapy in the fetus.

Here, we report the 18th patient (in the 14th publication) to receive prenatal transplacental sirolimus treatment for a fetus with a large, often life-threatening cardiac rhabdomyoma.

In our case, intrauterine progression of a cardiac mass with imminent bilateral outflow tract obstruction was observed. The combination of this obstruction risk and the high suspicion of rhabdomyoma—based on positive family history of genetically confirmed TSC (mother and sibling)—prompted the decision to start intrauterine mTOR-inhibitor therapy with sirolimus. Griesman et al. recently supported the initiation of transplacental targeted mTOR-inhibitor therapy for hemodynamically compromising cardiac tumors suspected to be rhabdomyomas, even when fetal genetic results are not yet available or are negative (18). We informed the parents about potential adverse effects of mTOR inhibitors, including transaminitis, proteinuria, hypertriglyceridemia, hyperlipidemia, diabetogenic effects, immunosuppression and infection, oral mucositis, pneumonitis, bone marrow suppression, and fetal growth restriction (Table 2) (33–37), as well as the off-label-use of sirolimus during pregnancy.

**Table 2:** Known side effects associated with mTOR-inhibitor therapy and their frequency (33–37).

Organ system	Adverse effect	Frequency (%)
Bone marrow/blood	Anemia	12–76
	Leucopenia	11
	Thrombocytopenia	Up to 30
Metabolism	Diabetes mellitus	20–27
	Hyperlipidemia	30–64
	Hypertriglyceridemia	21–57
	Hypercholesterolemia	20–46
Kidneys	Proteinuria	10
	Glomerulonephritis	2
	Thrombotic microangiopathy	Unknown
	Tubular toxicity	Unknown
Skin and mucosa	Oral ulceration	10–19
	Mucositis and stomatitis	3–8
Lung	Interstitial lung disease (clinical manifestation: dry cough, exercise dyspnea)	4–17
Intestine	Diarrhea	15–20
	Vomiting	
	Anorexia	
Vascular system	Angioedema	2.2–15
	Thromboembolic events	17
Lymphatic system	Lymphedema	6–12

We aimed for a maternal trough whole blood sirolimus level of 10–15 ng/dl, based on values reported in the literature (Table 1) (7–15) and in accordance with recommendations for adult transplant patients (38). Although the target trough sirolimus level was not reached, tumor progression rapidly stopped and outflow tract obstruction regressed under sirolimus treatment. However, treatment was discontinued due to the development of a progressive productive cough of unknown origin in the mother. Although cough is a commonly reported side effect of sirolimus, our main concern was the mother's increased risk of severe infection given her living and working on a farm, her mild intellectual impairment, and the potential risk of interstitial pneumonitis, a known but rare complication of sirolimus. Interstitial pneumonitis clinically manifests as dry cough and exertional dyspnea that develops over hours to days and may be accompanied by symptoms of hemoptysis and inflammatory syndrome. Diagnosis depends on imaging (radiographs and CT scan) and histology; however, the mother declined further diagnostic work-up. After stopping sirolimus, the cough promptly resolved. With the benefit of hindsight, we consider this to be a sirolimus-induced cough.

Despite the lack of risk factors other than sirolimus therapy, the mother developed gestational diabetes. After discontinuation of sirolimus, her blood glucose levels returned to the normal range. After birth, the decision to reinitiate mTOR-inhibitor therapy with everolimus in the neonate was based on the assumption that the perinatal re-entrant tachycardia was caused by residual tumor mass in the atrioventricular region. The massive capillary leak syndrome was likely caused by low cardiac output during tachycardia combined with restrictive ventricular filling (39). This condition may have been maintained by inotropic support, as adrenaline’s positive chronotropic effects shorten ventricular filling time. Everolimus may have further aggravated the situation, given that peripheral or generalized edema are common side effects (Table 2). Neither tachyarrhythmia nor arterial hypotension have been reported for everolimus or sirolimus to date. Considering these factors, neonatal everolimus therapy was continued with the aim of eliminating the presumed tumor remnant muscle bundle in the atrioventricular area thought to be substrate for re-entrant tachycardia (27, 28). The neonate's sirolimus blood level on the first day of life was <0.6 ng/ml. Unfortunately, the infant died before it was feasible to measure everolimus blood levels.

In conclusion, cardiac rhabdomyoma can pose a life-threatening risk to both fetus and neonate, not only by compromising myocardial function and cardiac outflow but also by causing arrhythmias through tumor muscle bundles that serve as substrates for pre-excitation syndromes, leading to intrauterine or postnatal atrioventricular re-entrant tachycardia (27, 28). When a tumor mass is located near the atrioventricular junction, careful and timely evaluation for (re)starting transplacental and neonatal mTOR-inhibitor therapy is crucial. Close monitoring of the newborn for arrhythmia development is mandatory. In addition, unfavorable maternal factors, such as immunosuppression, risk for infection, medication compliance, distance from the treating center, and living or working environments (e.g., remote farms), must be considered and warrant vigilant maternal monitoring, particularly in settings where there is an increased risk of infection. mTOR-inhibitor therapy for fetal cardiac rhabdomyoma is a tightrope walk between managing true therapeutic benefits and minimizing potential adverse effects.

## Data Availability

The original contributions presented in the study are included in the article/Supplementary Material, further inquiries can be directed to the corresponding author.
