# Correction: Drug review: mTOR-inhibitor therapy in fetal cardiac rhabdomyoma—a tightrope walk

**DOI:** 10.3389/fped.2025.1694368

**Published:** 2025-09-09

**Authors:** Nadine Muschel, Michaela Höck, Elke Griesmaier, Samira Abdel Azim, Elisabeth Ralser, Christina Schreiner, Elisabeth Schermer, Ursula Kiechl-Kohlendorfer, Irene Mutz-Dehbalaie, Miriam Michel

**Affiliations:** ^1^ Department of Obstetrics and Gynaecology, Medical University of Innsbruck, Innsbruck, Austria; ^2^ Department of Paediatrics II (Neonatology), Medical University of Innsbruck, Innsbruck, Austria; ^3^ Department of Paediatrics III (Cardiology, Pulmonology, Allergology and Cystic Fibrosis), Medical University of Innsbruck, Innsbruck, Austria

**Keywords:** adverse effects, fetal cardiac tumor, rhabdomyoma, sirolimus, re-entry tachycardia

The following **Acknowledgments** statement was erroneously omitted:

“The authors thank the patient and his family for their kind cooperation, and very much regret the child's adverse outcome. The authors thank E. Michel for manuscript proofreading and T. Klenner for creating the graphic and formatting the tables.”

The original version of this article has been updated.

